# Targeting the Unfolded Protein Response in Glioblastoma Cells with the Fusion Protein EGF-SubA

**DOI:** 10.1371/journal.pone.0052265

**Published:** 2012-12-20

**Authors:** Antony Prabhu, Bhaswati Sarcar, Soumen Kahali, Yuan Shan, Prakash Chinnaiyan

**Affiliations:** 1 Radiation Oncology, H. Lee Moffitt Cancer Center, Tampa, Florida, United States of America; 2 Experimental Therapeutics, H. Lee Moffitt Cancer Center, Tampa, Florida, United States of America; 3 Pathology, H. Lee Moffitt Cancer Center, Tampa, Florida, United States of America; 4 Cancer Imaging and Metabolism, H. Lee Moffitt Cancer Center, Tampa, Florida, United States of America; Ospedale Pediatrico Bambino Gesu’, Italy

## Abstract

Rapidly growing tumors require efficient means to allow them to adapt to fluctuating microenvironments consisting of hypoxia, nutrient deprivation, and acidosis. The unfolded protein response (UPR) represents a defense mechanism allowing cells to respond to these adverse conditions. The chaperone protein GRP78 serves as a master UPR regulator that is aberrantly expressed in a variety of cancers, including glioma. Therefore, cancer cells may be particularly reliant upon the adaptive mechanisms offered by the UPR and targeting GRP78 may represent a unique therapeutic strategy. Here we report that diffuse expression of GRP78 protein is present in Grade III-IV, but not Grade I-II glioma. To determine the role GRP78 plays in glioblastoma tumorigenesis, we explored the anti-tumor activity of the novel fusion protein EGF-SubA, which combines EGF with the cytotoxin SubA that has been recently shown to selectively cleave GRP78. EGF-SubA demonstrated potent tumor-specific proteolytic activity and cytotoxicity in glioblastoma lines and potentiated the anti-tumor activity of both temozolomide and ionizing radiation. To determine if the tumor microenvironment influences EGF-SubA activity, we maintained cells in acidic conditions that led to both UPR activation and increased EGF-SubA induced cytotoxicity. EGF-SubA was well tolerated in mice and led to a significant tumor growth delay in a glioma xenograft mouse model. The UPR is emerging as an important adaptive pathway contributing to glioma tumorigenesis. Targeting its primary mediator, the chaperone protein GRP78, through specific, proteolytic cleavage with the immunotoxin EGF-SubA represents a novel and promising multi-targeted approach to cancer therapy.

## Introduction

There are approximately 18,500 cases of newly diagnosed primary brain malignancies per year, with the most aggressive form, glioblastoma, being the most common [1]. Historically, achieving clinical gains in glioblastoma have been limited, however novel therapeutic strategies have emerged offering strong promise in this disease. In newly diagnosed glioblastoma, combining the alkylating agent temozolomide with radiation demonstrated a significant improvement in survival and now represents standard therapy in this malignancy [2]. Despite representing progress, this approach still does not offer cure to a majority of patients, who typically develop disease recurrence within a year of definitive therapy [1].

The recent focus of cancer drug development has been on highly specific therapies against purported molecular “drivers” of carcinogenesis. While these are generally well tolerated, clinical gains offered by these approaches have been limited, largely based on the complex nature of signaling networks associated with tumorigenesis and the inability to delineate the key “functional” signaling pathways actually driving growth in an individual tumor [3]. Regardless of the upstream “driving” signaling pathway, rapidly growing tumors require efficient means to allow them to adapt to fluctuating, toxic tumor microenvironments, which consist of hypoxia, nutrient deprivation, and acidosis. The unfolded protein response (UPR) represents a conserved, critical defense mechanism allowing cells to respond to these adverse conditions [4]. Therefore, cancer cells may be particularly reliant upon the adaptive mechanisms offered by the UPR for continued growth and survival in these otherwise cytotoxic conditions and modulating this adaptive response may represent a unique strategy for cancer therapy [5].

The chaperone protein glucose related protein 78 (GRP78), also referred to as the immunoglobulin binding protein (BiP), serves as a master UPR regulator that plays a central role in modulating its downstream signaling. Under non-stressed environmental conditions, GRP78 binds to its client proteins protein kinase RNA (PKR)-like ER kinase (PERK), activating transcription factor-6 (ATF6), and inositol-requiring protein-1 (IRE1). However, when the ER is “stressed”, GRP78 binds to the accumulating unfolded proteins in the ER, freeing its specific client proteins, leading to pathway activation [6]. Although regarded as a pro-survival mechanism, persistent or high-level activation of the UPR leads to apoptotic cell death [7], suggesting the potential for GRP78 to serve as a therapeutic target [5].

Several investigators have explored the potential for aberrant expression of UPR related proteins and their prognostic implications in cancer [8]. One of the first studies demonstrating this reliance of cancer cells on the UPR was presented by Jamora et al [9], in which GRP78-knockdown fibrosarcoma cells demonstrated similar *in vitro* growth characteristics as their parental line, however were not able to sustain growth *in vivo* in a mouse model. Since this discovery, several studies have validated the important role UPR related proteins play in tumorigenesis. Specific to glioma, Pyrko et al demonstrated that GRP78 is expressed at low levels in adult brain, but significantly elevated in malignant glioma and glioma cell lines [10]. Using microarray analysis, Lee et al similarly found that GRP78 expression was up-regulated in glioma and that its expression correlated with tumor grade [11]. Further, GRP78 expression had prognostic implications in glioblastoma, with increased expression portending poor survival. These studies also demonstrated that GRP78 contributed towards resistance to a variety of chemotherapeutics, including temozolomide, 5-fluorouracil, CPT-11, etoposide, cisplatin, and ionizing radiation [10], [11]. It has also been shown that GRP78 is highly elevated in the vasculature derived from human glioma specimens [12], [13] and powerfully regulates VEGF expression [14].

Selective destruction of GRP78 became possible with the discovery of a novel bacterial toxin SubA, which selectively cleaves only one protein, GRP78, at a single site, di-leucine motif (L416-L417) in the hinge region connecting the ATPase and protein-binding domains of the molecule [15]. GRP78 cleavage is rapid and virtually all intact GRP78 in the cell is degraded within 1–2 h of exposure, leading to massive apoptosis, even at toxin doses as low as 10 ng/mL, suggesting a highly potent catalytic activity [15]. To achieve selectively into cancer cells, we engineered a fusion protein epidermal growth factor (EGF)-SubA, combining EGF with SubA, which demonstrated significant inhibition of human breast and prostate cancer cells *in vitro* and *in vivo* [16]. Based on the clear biologic relevance the UPR and GRP78 play in glioma tumorigenesis [10], [11], we explored the anti-tumor potential of EGF-SubA in glioblastoma models.

## Materials and Methods

### Cell Culture

Human Glioblastoma cell lines U251, T98G and U87 were obtained from ATCC (Manassas, VA). U251 was grown in RPMI 1640 (GIBCO, Carlsbad, CA) supplemented with 5% heat inactivated fetal bovine serum. U87 and T98G were grown in Eagles minimum essential medium (ATCC, Manassas, VA), supplemented with 10% heat inactivated fetal bovine serum (GIBCO). Immortalized normal human astrocytes-SV40 (NHA) were obtained from Applied Biological Materials (ABM; Richmond, BC, Canada) and grown on Collagen IV (Sigma Aldrich; 2 mg/ml in 0.2% acetic acid) coated flasks or tissue culture plates in ABM Prigrow IV medium (ABM) supplemented with 10% heat inactivated fetal bovine serum (GIBCO). The glioblastoma neural stem (GNS) cell line G179 was provided by Dr. Austin Smith [17], distributed by BioRep (Milan, Italy), and grown in conditions as previously described [18]. All cells were grown in a humidified atmosphere at 37°C and 5% carbon dioxide. For acidic pH studies, respective media were acidified using 1N hydrochloric acid, pH tested, and filter sterilized. Cells were maintained in acidic conditions for at least 3 passages prior to performing the stated experiments.

### Treatment

The fusion protein EGF-SubA and control protein SubA lacking the targeting EGF moiety were provided by Sibtech, Inc. (Brookfield, Connecticut) as previously described [16] and dissolved in sterile PBS. Institutional safety guidelines were followed in handling the toxins. Temozolomide was purchased from Tocris Bioscience (Ellisville, MO) and dissolved in sterile DMSO. Cells were irradiated using the XRad 160 Xray source (Precision Xray Inc, N. Branford, CT) at 160 kV at a dose rate of 2.5 Gy/min.

### Clonogenic Assay

Cell survival was defined using a standard clonogenic assay. Cultures were trypsinized to generate a single-cell suspension and seeded into 6-well tissue culture plates. Irradiated feeder cells were used prior to U87 seeding to promote colony formation. Plates were then treated as described 16 h after seeding to allow cells to attach. Colonies were stained with crystal violet 10 to 14 d after seeding, the number of colonies containing at least 50 cells counted, and surviving fractions were calculated. Results were confirmed in three independent experiments.

### Immunoblot Analysis

Exponentially growing cells with or without treatment were lysed with ice-cold RIPA buffer (Sigma Aldrich) on ice. For *in vivo* studies, approximately 5 mg of flash frozen mouse brain, liver and tumor tissue were homogenized using a sterile Dounce homogenizer, suspended in 2 ml of ice cold RIPA buffer, and centrifuged at 8000 *g* for 10 m at 4°C. The supernatant was used for immunoblot analysis. Thirty µg of protein was resolved in 10% Tris-glycine SDS-PAGE and transferred to PVDF membrane (Millipore, Billerica, MA). The blots were probed with mouse anti-BiP/GRP78 (1∶10,000 BD Transduction Laboratories), mouse anti-β actin (1∶20,000 Sigma Aldrich), rabbit anti-PERK (1∶500, Cell Signaling), rabbit anti-phospho PERK (1∶1000, Santa Cruz Biotechnology), mouse anti-ATF6 (1∶1000, Abcam), rabbit anti-cleaved caspase 3 (1∶1000, Cell Signaling) and rabbit anti-EGFR (1∶1000, Abcam) antibodies. Anti-mouse or rabbit secondary antibodies conjugated with HRP was used for chemiluminescent detection (Thermo Fisher Scientific, Rockford, IL).

### *In-vivo* Tumor Growth

The University of South Florida Institutional Animal Care and Use Committee (IACUC) approved this study. Four to six week old athymic nu/nu mice (Charles River Laboratories) were used in the study. U251 cells (5×10^6^) were injected into the right hind flank subcutaneously. When the tumors reached a volume of ∼150 mm3 they were randomized into one of the two groups. One group received EGF-SubA (125 µg/kg; n = 6) in sterile PBS (100 µl) and the control group received the same volume of PBS alone (n = 6) subcutaneously behind the neck. A total of three doses were delivered every other day. The tumor volume (L x W x W/2) and mice weight were measured every other day. The mice were sacrificed when the tumor volume reached ≥1000 mm3. Prior to their tumors reaching this size, mice were euthanatized if they experienced an evidence of suffering, including inactivity, labored breathing, interfere with posture, locomotion or feeding, weight loss of more than 10%, or ulceration of the tumor. Mice were euthanatized by carbon dioxide.

### xCELLigence

Cell proliferation under normal and treated conditions were measured continuously using the xCELLigence System (Roche Diagnostics). The manufacturer’s protocol was followed. The proprietary 16 well plate was used for this purpose. A background reading of the plate was taken before seeding the cells. For G179 and NHA, the wells were coated with laminin and collagen, respectively. 10,000 cells in 100 µl of media were seeded in each well and placed in the instrument for measurement. A measurement was made every 15 minutes for the next 24 hours. Each well received 1 pM of EGF-SubA or SubA in 100 µl of media or pure media. The cells were monitored for the next 96 hours and the cell proliferation was measured as a cell index and plotted against time using proprietary software. Each treatment condition was measured as quadruplets and the mean cell index is represented. Results were confirmed in at least two independent experiments.

### Tissue Microarray

The glioma tissue microarray was purchased from US Biomax (Rockville, MD; GL 103a). The slides were stained using the Ventana Discovery XT Automated system (Ventana Medical Systems, Tuscon, AZ) following the manufacturer’s protocol with proprietary reagents. The slides were deparaffinized and a heat induced antigen retrieval protocol was followed using a Ribo CC buffer (Ventana). The array was stained with rabbit anti-Bip/GRP78 antibody (1∶200; Abcam, Cambridge, MA) diluted with Dako antibody diluent (Carpenteria, CA) for 32 minutes. The slides were incubated in Ventana omniMap anti-rabbit secondary antibody for 20 minutes. The slides were counter stained with hematoxylin and detected with Ventana ChromoMap Kit. The neuropathologist confirmed the histology of all samples and was blinded to grade when determining the expression level of GRP78 in tumors.

### Reverse Transcriptase PCR Analysis

Total cellular RNA was isolated using the Qiagen RNeasy kit (Qiagen, Valencia CA). Transcript level of XBP1, GRP78 and GAPDH mRNA were analyzed using 500 ng of total RNA. TaKaRa RNA PCR kit (Takara Bio USA, Madison, WI) was used for this purpose. Bip/GRP78 primer pairs: GRP78-F, 5′- TGCAGCAGGACATCAAGTTC-3′, and GRP78-R, 5′- CGCTGGTCAAAGTCTTCTCC-3′, amplicon size 460 bp. Xbp1 primer pairs: Xbp1-F, 5′-GTTGAGAACCAGGAGTTAAGACAG-3′, Xbp1-R, 5′-CAGAGGGTATCTCAAGACTAGG-3′. Activation of Ire1 following UPR activation was measured by the splicing of mRNA encoding XBP1. A 456 bp and 430 bp PCR product is expected if the XBP1 amplicon is derived from the unspliced and spliced form, respectively. GAPDH primer pairs: GAPDH-F, 5′-CTCAGACACCATGGGGAAGGTGA-3′, GAPDH-R, 5′-ATGATCTTGAGGCTGTTGTCATA-3′ amplicon size 450 bp. PCR was performed by denaturing at 94°C for 1 m, annealing at 55 °C for 1 m, elongation at 72 °C for 1 m for a total of 30 cycles, with a final extension step at 72 °C for 7 m for all amplifiable products. The PCR products were resolved in a 2.5% agarose gel and visualized under UV light.

### Statistics

The statistical analysis was done for the described treatment conditions using a Student’s *t* test. A probability level of a *P* value of <0.05 was considered significant.

## Results and Discussion

**Figure 1:**
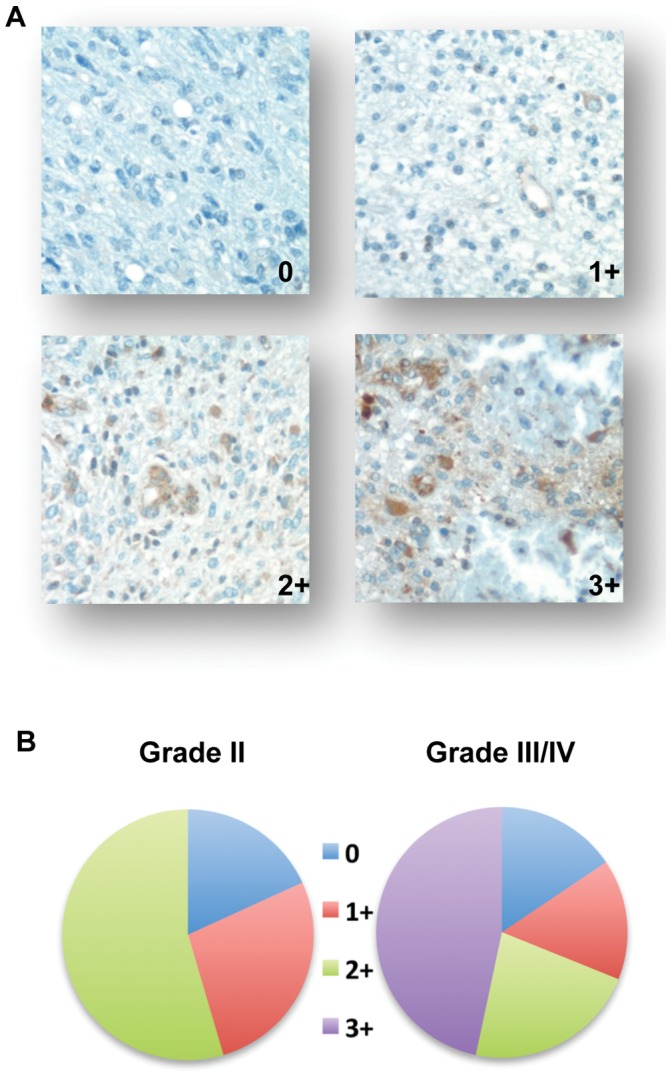
GRP78 expression in glioma. Immunohistochemical staining was performed on a glioma tissue microarray using an anti-GRP78 antibody and expression levels (0, 1+, 2+, and 3+) were quantified based on the intensity of staining. Representative staining patterns (A) and tumor grade-specific distributions of identified staining intensities (B) are provided.

Previous investigations have demonstrated aberrant expression of GRP78 in malignant glioma at the transcriptional level [10], [11], although its expression at the protein level has yet to be comprehensively quantified. We therefore performed immunohistochemical staining on a glioma tissue microarray (TMA). The TMA consisted of a total of 56 glioma specimens, 11 samples were Grade II and 45 samples were Grade III/IV; individual histologies are provided in Fig. S1. The expression level of GRP78 ranged from no expression (0) to diffuse expression (3+), with representative images provided in Fig. 1A. Although various levels of focal GRP78 expression was present in the different grades of glioma, diffuse expression (3+) was only present in Grade III and IV tumors (n = 21/45), further supporting the biologic relevance the UPR plays in malignant glioma and its potential to serve as a molecular target.

**Figure 2:**
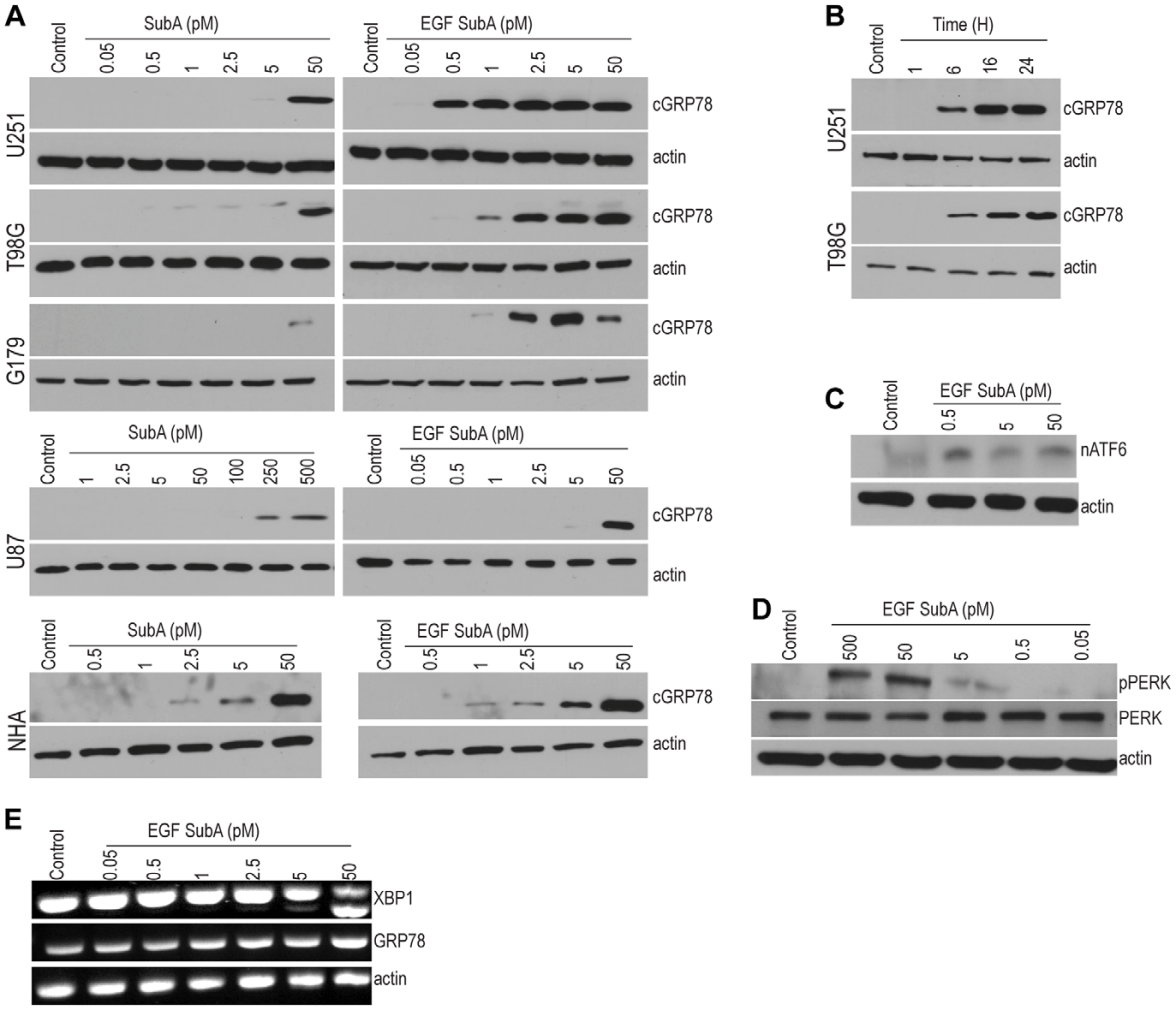
SubA and EGF-SubA cleaves GRP78 and activates the UPR. Exponentially growing glioblastoma cell lines and normal human astrocytes (NHA) were (A) treated with SubA or EGF-SubA at the specified picomolar concentrations for 24 h or (B) exposed EGF-SubA (1 pM) for the specified time periods. Total cellular protein was isolated and immunoblotting was performed with anti-GRP78 antibody. SubA and EGF-SubA cleaved the endogenous GRP78 (78 kDa) resulting in an additional smaller fragment of 28 kDa (cGRP78). (C-E) Total cellular protein and RNA were isolated from U251 cells exposed to EGF-SubA at the stated concentrations for 24 h. EGF-SubA induced GRP78 cleavage resulted in nuclear localization of ATF6 (C; nATF6), a dose-dependent phosphorylation of PERK (D; pPERK), and Ire1 activation, determined by Xbp1 mRNA splicing (E). Each figure is a representative of three independent experiments.

We have previously demonstrated selective cleavage of GRP78 in EGFR-positive prostate and breast cancer cells exposed to EGF-SubA; thereby confirming the receptor-binding activity of the EGF moiety and the proteolytic activity of the SubA moiety [16]. We now extend these studies to explore the potential of both the fusion protein EGF-SubA and the SubA toxin alone to cleave GRP78 in glioblastoma models. As demonstrated in Fig. 2A, EGF-SubA demonstrated potent proteolytic activity, cleaving GRP78 at concentrations ranging from 0.5 to 2.5 pM in established glioblastoma cell lines (U251 and T98G) and the glioblastoma neural stem (GNS) cell line G179. These concentrations were over 20 fold lower when compared to the SubA toxin alone, which required approximately 50 pM to induce GRP78 cleavage, confirming increased potency of the fusion protein EGF-SubA. Time course studies demonstrated maximal cleavage of GRP78 within 16 h of EGF-SubA exposure (Fig. 2B). Conversely, cleavage of GRP78 in normal human astrocytes (NHA) required significantly higher concentrations of EGF-SubA when compared to the glioblastoma cell lines, supporting the tumor specificity of this approach. Interestingly, the glioblastoma cell line U87 required considerably higher concentrations of EGF-SubA and SubA toxin to induce GRP78 cleavage. As an initial investigation, based on the mechanism of action of EGF-SubA, we performed western blot analysis to determine if the relative expression of EGFR or GRP78 could contribute to the observed differential response of EGF-SubA. As demonstrated in Fig. S2, expression levels of these proteins did not appear to be significantly different between the cancer cell lines tested. As we reported earlier, EGF-SubA induced toxicity is EGFR-dependent, but does not directly correspond to the EGFR expression level, reflecting a more complex cell-specific process of EGFR-mediated internalization and trafficking, as well as the magnitude of ER stress and UPR signaling in a particular cell line^16^. Therefore, the factors contributing towards relative sensitivity and resistance to EGF-SubA remain an active area of investigation.

**Figure 3:**
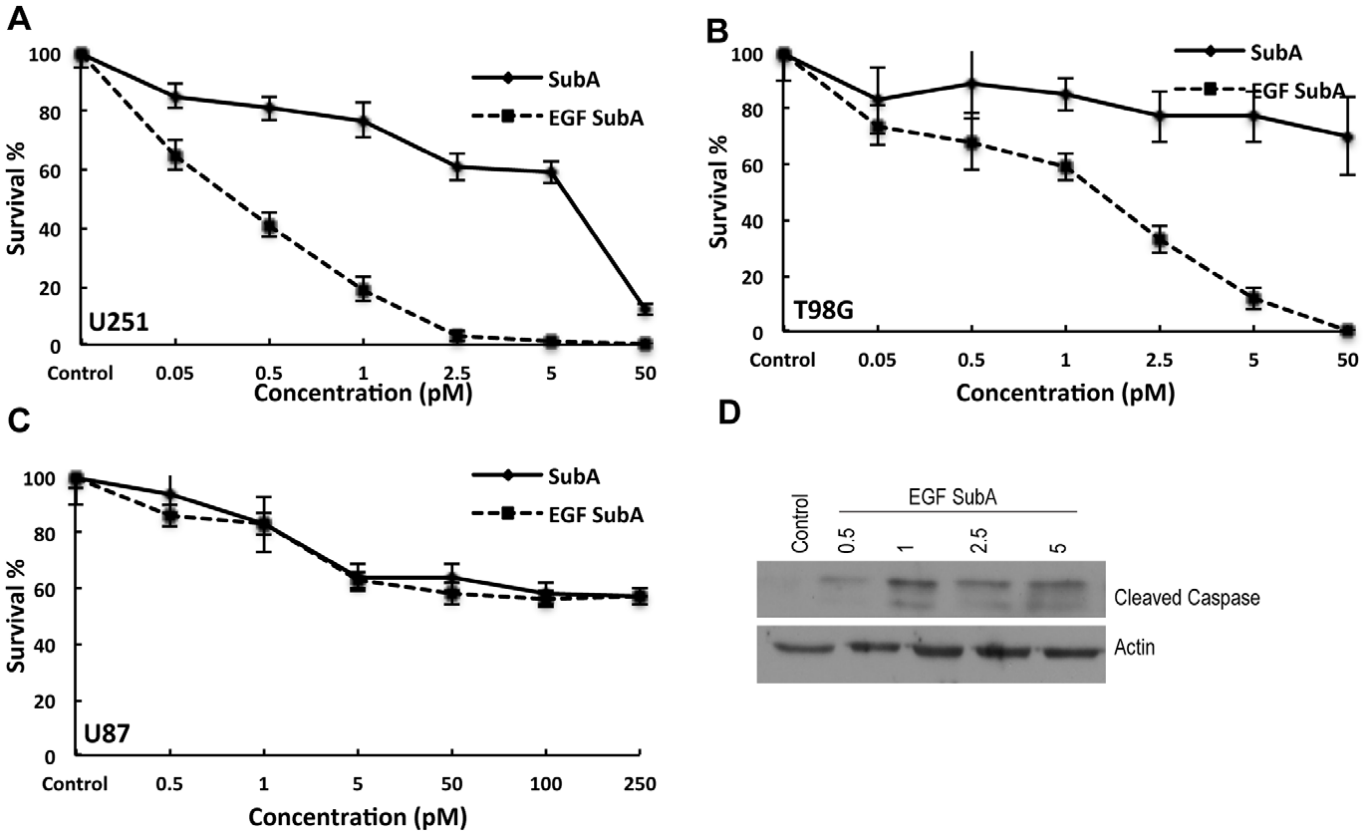
The influence of SubA and EGF-SubA on glioma cell survival. A clonogenic assay was performed to study the cytoxicity of SubA and EGF-SubA in U251 (A), T98G (B) and U87 cells (C). Cells were seeded as single cell suspensions in six well culture plates, allowed to adhere, and treated with the stated concentrations of SubA or EGF-SubA for 24 h. Plates were then replaced with fresh culture media and surviving fractions were calculated 10 to 14 d following treatment. Cell survival was significantly different between SubA and EGF SubA treatment in U251 (p<0.0001) and T98G (p<0.0001 at concentrations ≥0.5 pM) and not significant in U87 cells (p = 0.2112). (D) Immunoblotting of total cellular protein from U251 cells treated with EGF-SubA at the stated concentrations for 24 h demonstrates EGF-SubA induced apoptosis, as determined by cleaved caspase 3. Each figure is a representative of three independent experiments.

We went on to determine the influence of EGF-SubA induced cleavage of GRP78 on UPR activation. As describe above, the primary three mediators involved in UPR signaling include PERK, Ire1, and ATF6. Upon stress, PERK is released from GRP78 to permit homodimerization, autophosphorylation and pathway activation. Similarly Ire1 is activated by dimerization, leading to trans-autophosphorylation; however, pathway activation does not entail a conventional cascade of sequential kinase activation, rather, activation of a cytosolic endoribonuclease activity whose only know substrate is X-box binding protein-1 (Xbp1) mRNA. This alters the Xbp1 translational reading frame leading to activation of a unique UPR specific program. The third mediator, ATF6, is concomitantly released from GRP78, permitting its transport to the Golgi compartment where it is cleaved to generate the cytosolic activated form of ATF6 that translocates to the nucleus [4], [6]. In our studies, all three pathways were activated in U251 cells following exposure to EGF-SubA, as determined by PERK phosphorylation (Fig. 2D), nuclear localization of cleaved ATF6 (Fig. 2C), and splicing of Xbp1 mRNA (Fig. 2E). However, the EGF-SubA concentrations required to induce Xbp1 splicing were significantly higher than what was demonstrated to induce GRP78 cleavage (Fig. 2A) and cytotoxicity (Fig. 3A); therefore, these findings suggest that this pathway does not play a significant role in the observed anti-tumor activity of EGF-SubA.

Next, the cytotoxicity of EGF-SubA and SubA were evaluated in these models using a clonogenic assay. In these studies, the respective glioblastoma cell lines were plated as singe cells, and exposed to either EGF-SubA or SubA for 24 h; culture plates were then replaced with fresh media and placed back into the incubator to allow for colony formation. As demonstrated in Fig. 3, EGF-SubA demonstrated potent cytotoxicity, with IC_50_ values corresponding to the concentrations required for GRP78 cleavage, ranging from 0.5 pM (in U251) to 2.5 pM (in T98G; Fig. 3 A/B). Importantly, these concentrations were several orders of magnitude more potent than SubA toxin alone, which again corresponds to the increased ability of the fusion protein to target and cleave GRP78. Furthermore, U87 cells demonstrated relative resistance to EGF-SubA cytotoxicity when compared to the other lines (Fig. 3C), as predicted by its limited capacity of cleaving GRP78 in this specific line. Western blot was performed to define the mode of cell death following EGF-SubA. As demonstrated in Fig. 3D, exposing U251 cells to EGF-SubA for 24 h lead to an increase in apoptosis, as determined by cleaved caspase.

**Figure 4:**
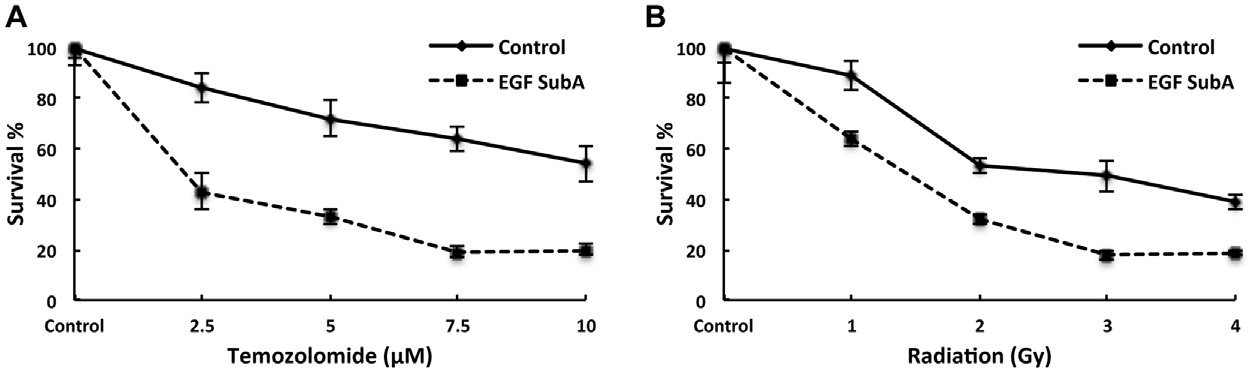
EGF-SubA enhances anti-tumor activity of temozolomide and ionizing radiation. A clonogenic assay was performed to evaluate the potential of EGF-SubA to enhance temozolomide (A) (statistically significant p<0.0001) and radiation-induced (B) cytotoxicity (statistically significant p<0.0024). U251 cells were seeded in six well culture plates and exposed to 1 pM of EGF-SubA 16 h prior to the addition of temozolomide or radiation exposure. Fresh media was then replaced in the culture plates after 8 h, and surviving fractions were calculated 10 to 14 d following treatment, normalizing for the individual cytotoxicity of EGF-SubA. Each figure is a representative of three independent experiments.

As GRP78 has been previously reported to contribute towards therapeutic resistance [5], [8], [10], [11], [12], [13], [19], we next examined the potential of EGF-SubA to enhance the anti-tumor activity of standard cytotoxics in glioblastoma, including temozolomide and ionizing radiation [1]. In these experiments, U251 cells were exposed to EGF-SubA (1.0 pM) 16 h prior to either temozolomide or ionizing radiation. As demonstrated in Fig. 4, in addition to potent independent activity, EGF-SubA demonstrated the capacity to enhance both temozolomide-induced cytotoxicity (Fig. 4A) and response to therapeutic doses of ionizing radiation (Fig. 4B), further supporting this strategy in the treatment of glioblastoma.

**Figure 5:**
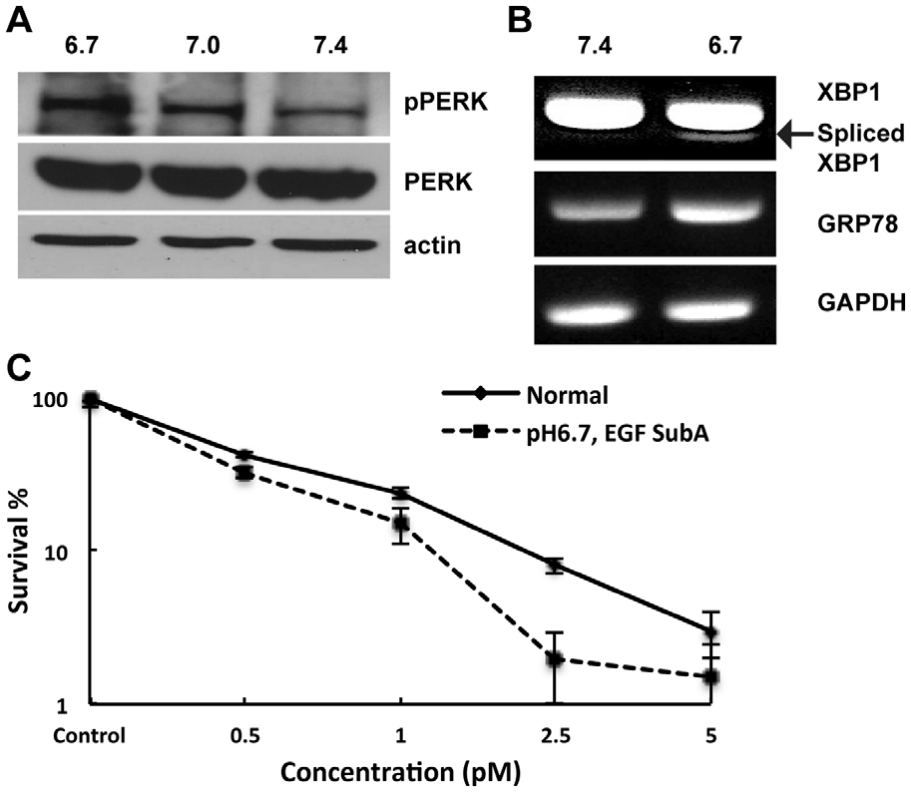
Acidic pH activates the UPR pathway and enhances EGF-SubA cytotoxicity. U251 cells grown in RPMI media whose pH was adjusted to 6.7 and 7.0 with 1N HCl for 3 passages prior to performing experiments demonstrated UPR activation, as determined by PERK phosphorylation (A; pPERK), Xbp1 splicing and increased GRP78 transcription (B). (C) To determine if cells grown in acidic conditions influenced EGF-SubA cytotoxicity, a clonogenic assay was performed with U251 cells grown in normal (pH 7.4) or acidic (pH 6.7) conditions at the stated concentrations. Cell survival was significantly different between cells grown in normal and acidic pH at higher doses of EGF SubA (p<0.0001 at 2.5 pM). Each figure is a representative of three independent experiments.

As described above, the UPR represents an important adaptive process that allows cells to survive microenvironmental stresses, including hypoxia, acidosis, and nutrient deprivation [4]. Although cells growing in such conditions have been previously associated with therapeutic resistance [20], we hypothesized that they would be more reliant on the UPR for survival, and therefore, particularly sensitive to UPR modulation. As an initial investigation, we studied the role acidosis may play in UPR activation [21]. U251 cells serially maintained in acidic conditions (pH 6.7) demonstrated UPR activation when compared to cells grown in standard media (pH 7.4), including PERK phosphorylation (Fig. 5A), Xbp1 splicing, and increased GRP78 transcription (Fig. 5B). Further, as we hypothesized, U251 cells grown in acidic conditions demonstrated increased sensitivity to EGF-SubA cytotoxicity, as determined by the clonogenic assay (Fig. 5C).

In an effort to evaluate the cytotoxicity of EGF-SubA in both normal human astrocytes and GNS cells, which have limited capacity of growing as viable colonies, we applied the xCELLigence system, which allows for a real-time, label-free analysis of cellular growth by monitoring electrical impedance using specialized culture plates [22]. As an initial investigation, we sought to first confirm the anti-tumor activity of EGF-SubA in U251 cells using this platform. Continuous exposure of U251 cells to 1.0 pM of EGF-SubA, which represents a concentration that led to significant cytotoxicity in the clonogenic assay (Fig. 3A), demonstrated a similarly potent anti-tumor activity on the xCELLigence platform (Fig. S3A). In addition, as this assay was performed in real-time, we were able to identify that EGF-SubA induced cytotoxicity began approximately 8 h following exposure, which corresponds to the observed temporal dynamics of GRP78 cleavage presented in Fig. 2B, further supporting its underlying mechanism of action. Interestingly, as opposed to U251 controls, in which surviving cell populations quickly resumed proliferation, U251 cells grown in acidic conditions (pH 6.7) maintained an attenuated repopulation, supporting our previous findings of increased cellular sensitivity to EGF-SubA in acidic conditions. We then extended this assay to the GNS cell line G179 and normal human astrocytes. Similar to U251, G179 cells also demonstrated potent cytotoxicity of EGF-SubA (1.0 pM) when compared to SubA toxin alone and attenuated repopulation in cells grown in acidic conditions (Fig. S2B). To support the therapeutic potential of this approach, we did similar studies using normal human astrocytes. As shown in Fig. S2C, EGF-SubA (1.0 pM) demonstrated no activity in human astrocytes, which corresponds to our previous findings suggesting higher concentrations of EGF-SubA would be required to induce GRP78 cleavage (Fig. 2A).

**Figure 6:**
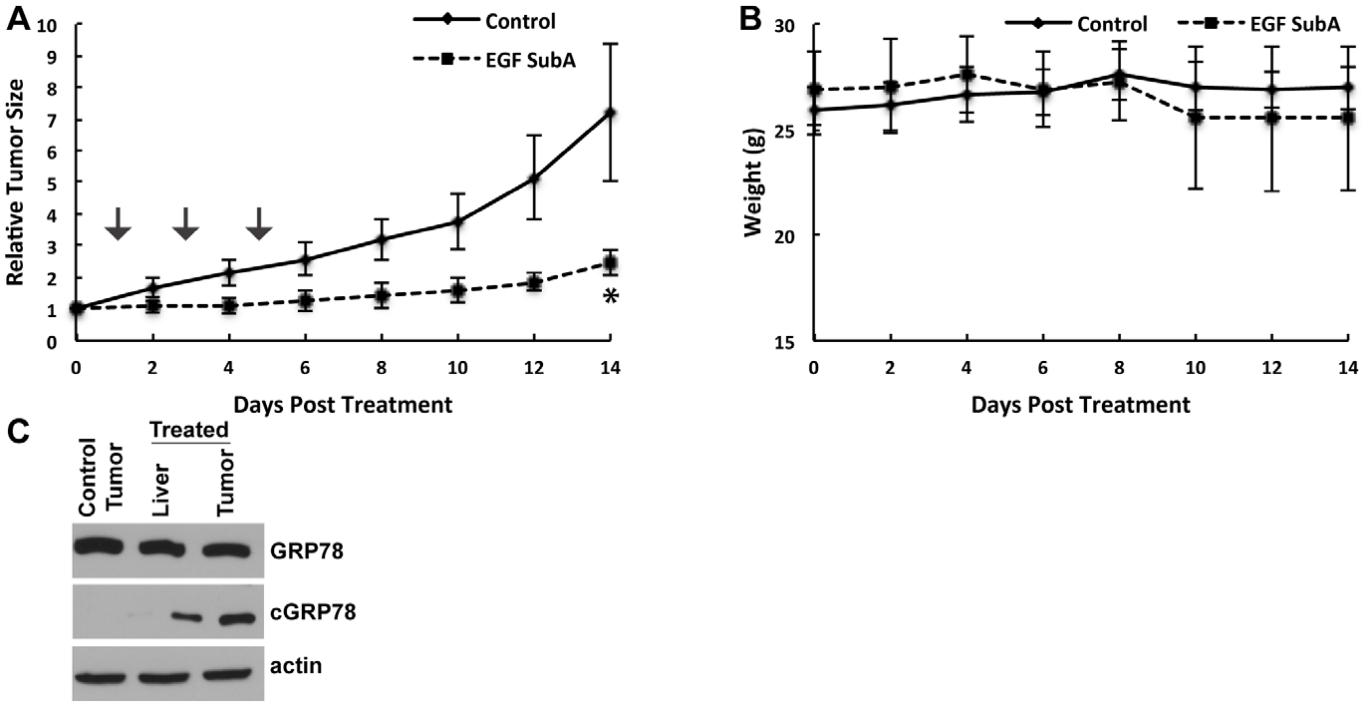
EGF-SubA delays tumor growth in mice. U251 cells were injected s.c in a mouse flank model (A). When tumors reached ∼150 mm^3^ in size, mice were randomized into two groups: vehicle control (PBS) or EGF-SubA (125 µg/kg) delivered s.c. on the stated days (arrow). To obtain a tumor growth curve, perpendicular diameter measurements of each tumor were measured with digital calipers, and volumes were calculated using the formula (*L* × *W* × *W*)/2. Tumor volumes (A) and weight of mice (B) were measured every other day. Tumor volumes were normalized to their volume at randomization. Each group contained six mice. *p = 0.0009. (C) U251 cells were injected s.c. in a mouse flank model. When tumors reached ∼500 mm^3^ in size, mice were exposed to either PBS alone or EGF-SubA (125 µg/kg). Mice were then sacrificed 24 h after treatment and stated tissue was dissected, flash frozen, and tissue lysates were generated to evaluate for GRP78 cleavage by immunoblot.

Lastly, we extended our *in vitro* findings *in vivo* using a mouse xenograft model. U251 cells were implanted s.c. into the hind leg of nude mice and randomized to control (PBS) or EGF-SubA (125 ug/kg) delivered s.c. every other day for 3 days. As demonstrated in Fig. 6A, although this approach did not result in any notable tumor regression, a significant growth delay was observed with EGF-SubA (*p* = 0.0009). In addition, this regimen was well tolerated, demonstrating no significant weight loss in EGF-SubA treated mice (Fig. 6B; *p* = 0.47). Next, to confirm *in vivo* target engagement of EGF-SubA and to evaluate for potential normal tissue toxicity of this compound, we performed western blot on tissue lysates 24 h following EGF-SubA treatment. As demonstrated in Fig. 6C, GRP78 was expressed in U251 tumors and in mouse liver. Consistent with *in vitro* data, EGF-SubA cleaved GRP78 in U251 tumors grown subcutaneously. Normal liver cells express EGFR; therefore as expected, there was modest GRP78 cleavage observed in the mouse liver, although it was not associated with any significant weight loss or activity. This finding is consistent with the previous report that up to 50% decrease in GRP78 expression does not affect physiologically normal organs and tissues, however significantly impedes tumor growth and angiogenesis [23]. Nevertheless this may represent a potential dose-limiting toxicity of this compound.

In summary, the UPR is emerging as an important adaptive pathway contributing to malignant glioma survival. Targeting its primary mediator, the chaperone protein GRP78, through specific, proteolytic cleavage with the immunotoxin EGF-SubA represents a novel and promising multi-targeted approach to cancer therapy. Our work confirms the potential of GRP78 to serve as a molecular target in malignant glioma and demonstrates potent tumor specific cytotoxicity of EGF-SubA in a panel of glioblastoma models *in vitro* and *in vivo*. These findings provide the framework for further investigations designed to target the UPR in glioma.

## Supporting Information

Figure S1 **Samples Histology and densitometry.** Histology of samples used in the glioma tissue microarray (A). Densitometry analysis of Western blots (B).
(TIF)

Figure S2 **EGFR and GRP78 expression in tumor cells.** Depicted cell lines were lysed and total cellular protein isolated to evaluate for (A) EGFR and (B) GRP78 expression by immunoblot. Each figure is a representative of three independent experiments.
(TIF)

Figure S3 **Effect of EGF SubA and SubA on cell proliferation.** The influence of EGF-SubA and SubA on cell proliferation in the described cell lines and conditions were measured in real time by the xCELLigence system. Cells were seeded in proprietary plates and exposed to 1 pM of SubA, EGF-SubA, or PBS alone. Cell proliferation was monitored every 15 minutes and the results are represented as the mean of quadruplets for each assay condition. This figure is a representative of three independent experiments.
(TIF)
