# Supplementary figures and images for: Targeting the Unfolded Protein Response in Glioblastoma Cells with the Fusion Protein EGF-SubA
Source: PLoS One. 2012 Dec 20;7(12):e52265. doi: 10.1371/journal.pone.0052265 (PMC3527517; doi:10.1371/journal.pone.0052265)

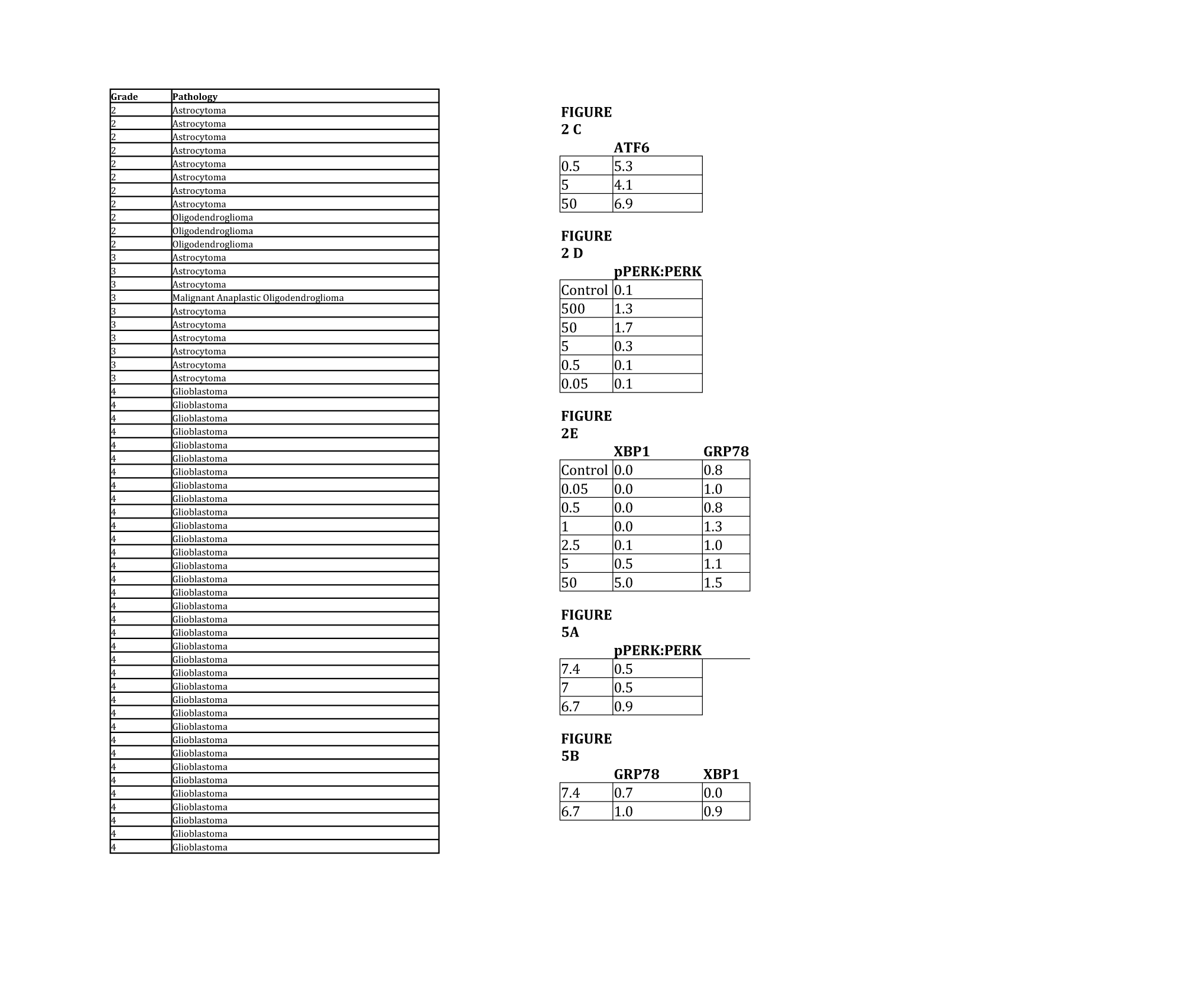

Supplement: Figure S1 — Samples Histology and densitometry. Histology of samples used in the glioma tissue microarray (A). Densitometry analysis of Western blots (B). (TIF) [file pone.0052265.s001.tif]

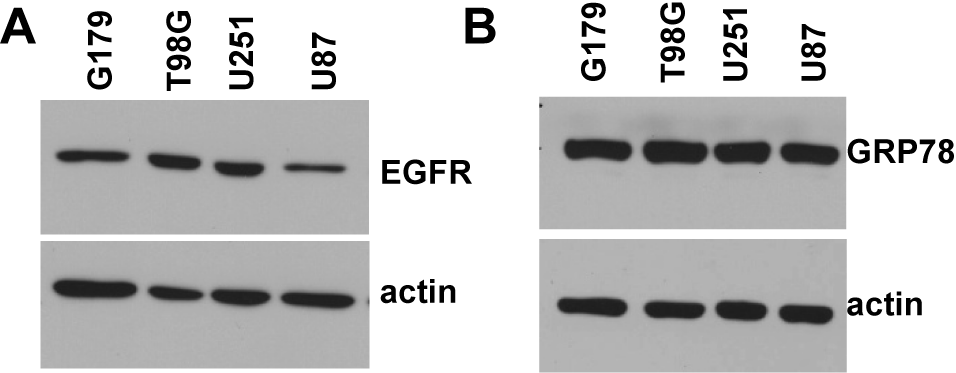

Supplement: Figure S2 — EGFR and GRP78 expression in tumor cells. Depicted cell lines were lysed and total cellular protein isolated to evaluate for (A) EGFR and (B) GRP78 expression by immunoblot. Each figure is a representative of three independent experiments. (TIF) [file pone.0052265.s002.tif]

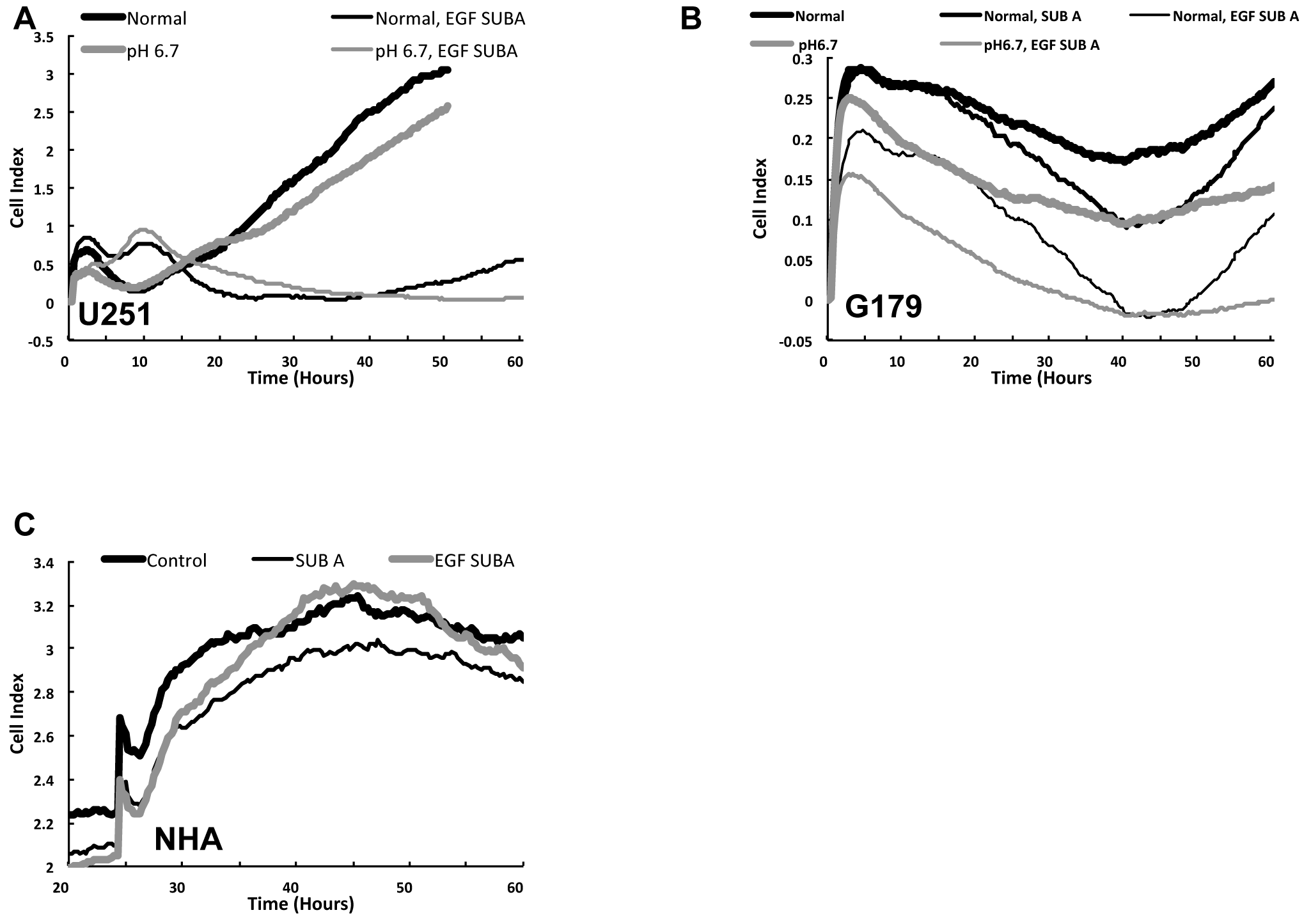

Supplement: Figure S3 — Effect of EGF SubA and SubA on cell proliferation. The influence of EGF-SubA and SubA on cell proliferation in the described cell lines and conditions were measured in real time by the xCELLigence system. Cells were seeded in proprietary plates and exposed to 1 pM of SubA, EGF-SubA, or PBS alone. Cell proliferation was monitored every 15 minutes and the results are represented as the mean of quadruplets for each assay condition. This figure is a representative of three independent experiments. (TIF) [file pone.0052265.s003.tif]
